# Distinct Effects of Guanidine Thiocyanate on the Structure of Superfolder GFP

**DOI:** 10.1371/journal.pone.0048809

**Published:** 2012-11-07

**Authors:** Olesya V. Stepanenko, Olga V. Stepanenko, Irina M. Kuznetsova, Daria M. Shcherbakova, Vladislav V. Verkhusha, Konstantin K. Turoverov

**Affiliations:** 1 Laboratory of Structural Dynamics, Stability and Folding of Proteins, Institute of Cytology of the Russian Academy of Sciences, St. Petersburg, Russia; 2 Department of Anatomy and Structural Biology, Albert Einstein College of Medicine, New York, New York, United States of America; The Beatson Institute for Cancer Research, United Kingdom

## Abstract

Having a high folding efficiency and a low tendency to aggregate, the superfolder GFP (sfGFP) offers a unique opportunity to study the folding of proteins that have a β-barrel topology. Here, we studied the unfolding–refolding of sfGFP that was induced by guanidine thiocyanate (GTC), which is a stronger denaturing agent than GdnHCl or urea. Structural perturbations of sfGFP were studied by spectroscopic methods (absorbance, fluorescence, and circular dichroism), by acrylamide quenching of tryptophan and green chromophore fluorescence, and by size-exclusion chromatography. Low concentrations of GTC (up to 0.1 M) induce subtle changes in the sfGFP structure. The pronounced changes in the visible absorption spectrum of sfGFP which are accompanied by a dramatic decrease in tryptophan and green chromophore fluorescence was recorded in the range 0–0.7 M GNC. These alterations of sfGFP characteristics that erroneously can be mixed up with appearance of intermediate state in fact have pure spectroscopic but not structural nature. Higher concentrations of GTC (from 0.7 to 1.7 M), induce a disruption of the sfGFP structure, that is manifested in simultaneous changes of all of the detected parameters. Full recovery of native properties of denaturated sfGFP was observed after denaturant removal. The refolding of sfGFP passes through the accumulation of the off-pathway intermediate state, in which inner alpha-helix and hence green chromophore and Trp57 are still not tuned up to (properly integrated into) the already formed β-barrel scaffold of protein. Incorporation of the chromophore in the β-barrel in the pathway of refolding and restoration of its ability to fluoresce occur in a narrow range of GTC concentrations from 1.0 to 0.7 M, and a correct insertion of Trp 57 occurs at concentrations ranging from 0.7 to 0 M GTC. These two processes determine the hysteresis of protein unfolding and refolding.

## Introduction

**Figure 1:**
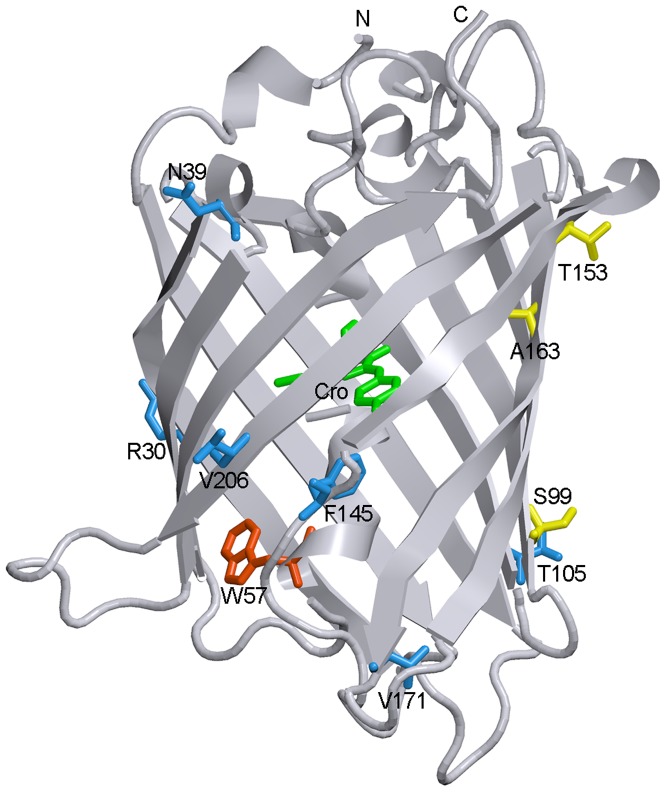
Spatial structure of sfGFP. The localizations of chromophore (green) and single tryptophan residue Trp 57 (red) are shown. Six amino acids which were mutated in the 'cycle-3' GFP variant to yield sfGFP (S30R, Y39N, N105T, Y145F, I171V and A206V) are drawn as blue stick unions. The amino acids corresponding to the 'cycle-3' mutations F99S, M153T and V163A are shown as yellow stick unions. The figure is created on the basis of PDB data [38] with the files 2B3P.ent for sfGFP [35], using the graphical software VMD [69] and Raster 3D [70].

Fluorescent proteins (FPs) are small proteins with barrel-fold topology. A unique chromophore of FPs, which originated from three intrinsic amino acids in positions 65–67, is tightly encapsulated inside the barrel and does not require any cofactors or enzymatic systems to be formed, except for molecular oxygen [1]. The rigid β-barrel shell of FPs performs important functions [2], [3], protecting a protein chromophore from any environmental factors and from radiationless deactivation while it restricts chromophore flexibility (Fig. 1). The correct folding of the protein matrix is strongly obligated for chromophore maturation because it results in proper orientation of the amino acids that catalyze chromophore synthesis. Protein folding provides a bend in the central α-helix that bears the chromophore-forming tripeptide, which is required for chromophore synthesis.

Many applications of FPs in the field of modern biology are based on the diversity of currently available FPs [2], [4], [5], [6], [7]. A wide palette of FP colors overlaps almost all visible spectra regions. FPs have been used for highlighting of cellular processes and for the localization and dynamics of single proteins, cellular organelles and whole cells [8], [9]. Genetic incorporation of FP pairs into FRET (Forster resonance energy transfer) biosensor systems has allowed the monitoring of protein-protein interactions, enzymatic activities and concentrations of metabolites in live cells [4], [10]. FPs that are sensitive to surrounding factors, such as pH, ion composition or redox potential, have been applied to single FP-based biosensor systems [11], [12], [13], [14], [15]. Recent developments of FPs with light-modulated spectral properties, far-red FPs and FPs with a large Stokes shift have enabled the imaging of fixed and live cells using super-resolution imaging techniques and the visualization of cellular processes in animals [5], [16], [17], [18], [19]. Notably, the diversification of biochemical and photophysical properties of FPs is achieved not only through a variety of chromophore structures but also through the tuning of contacts of a chromophore with the barrel matrix surrounding it [16], [20], [21], [22], [23], [24].

Because the proper fold of β-barrels underlies the acquisition of FP functions (i.e., the ability to fluoresce), the study of the pathways of FP folding appears to be important. Moreover, insight into this problem will enrich our knowledge of the fundamental question of the folding of proteins with β-barrel topology. In these types of studies, a chromophore serves as a marker of the integrity of the protein structure because it has a high quantum yield when it is submerged in a rigid FP barrel [25], [26]. The recent studies of folding processes and stability of many FPs showed that these proteins are characterized by complex energy landscape with high energy barriers and a number of different intermediate states [27], [28], [29], [30], [31], [32]. These studies are summarized in a thorough review [33]. A distinctive feature of FPs unfolding – refolding processes is apparent hysteresis of unfolding – refolding processes [30]. Though it was proposed that extremely slow unfolding and refolding processes are connected with isomerization of proline residues which are numerous in FP [29], [30], the main role belongs to the chromophore [31]. Indeed, FP mutant variants defective of chromophore formation showed no hysteresis of the unfolding and refolding curves [29]. Thus, once being formed chromophore of FPs plays a crucial role in protein stability and folding. Another characteristic feature of FPs is their high resistancе to different denaturing actions. Therefore, to probe unfolding and refolding processes of FPs we chose guanidine thiocyanate (GTC) which is known to have stronger denaturanting action in comparison with guanidine hydrochloride (GdnHCl) or urea and has not been applied to FP studies before.

Superfolder GFP variant (sfGFP) was selected as an object of investigation. This protein does not aggregate and has a strong tendency toward refolding, in contrast to other FPs [34], [35]. These properties make sfGFP a preferable object for the study of the processes of folding and unfolding in FPs. We observed two sub-ranges of GTC concentrations in which the effects of this denaturing agent on the protein structure are different. At moderate GTC concentrations, the overall sfGFP structure remains unchanged, though due to result of protein molecules interaction with denaturant molecules the visible absorption spectrum of the protein is significantly altered and hence thryptophan and chromophore fluorescence intensity change. We supposed that chromophore inside β-barrel scaffold tightens β-barrel through non-covalent interaction with surrounding protein matrix thus elevating protein stability. The refolding of sfGFP passes through the accumulation of the off-pathway intermediate state, in which inner alpha-helix and hence green chromophore and Trp57 are still not tuned up to (properly integrated into) the already formed β-barrel scaffold of protein.

## Materials and Methods

### Gene Expression and Protein Purification

The plasmid pET-28a (+)-sfGFP encoding for superfolder GFP [35] with poly-histidine tag was constructed as described previously [36] and was transformed into an *Escherichia coli* BL21(DE3) host (Invitrogen). The sfGFP expression was induced by an incubation of the cells with 0.5 mM isopropyl-beta-D-1-thiogalactopyranoside (IPTG; Fluka, Switzerland) during 24 h at 23°C. Recombinant protein was purified with Ni+-agarose packed in His-GraviTrap columns (GE Healthcare, Sweden). The purity of the protein was not less than 95%, as indicated by SDS-PAGE in 15% polyacrylamide gel [37]. Optical density of protein samples does not exceed 0.2. Measurements were performed in 50 mM TrisCl buffer, pH 8.0.

### Spectrophotometric Experiments

Absorption spectra were recorded using an EPS-3T spectrophotometer (Hitachi, Japan). The experiments were performed in microcells 101.016-QS 5 mm×5 mm (Hellma, Germany) at room temperature.

### Analysis of Protein 3D Structure

An analysis of the microenvironment peculiarities of the tryptophan and tyrosine residues localized in the structure of proteins was performed on the basis of PDB data [38], using the 2B3P.ent file [35] for sfGFP and the 2Y0G.ent file [39] for EGFP. The analysis of the peculiarities of the microenvironment of the tryptophan and tyrosine residues localized in the protein was performed as described previously [40], [41], [42], [43].

### Fluorescence Spectroscopy

Fluorescence experiments were conducted using a Cary Eclipse spectrofluorometer (Varian, Australia) with microcells FLR (10×10 mm; Varian, Australia). Fluorescence anisotropy and fluorescence lifetime were measured using homebuilt spectrofluorometers with steady-state and time-resolved excitation [44] using micro-cells (101.016-QS 5×5 mm; Hellma, Germany).

The tryptophan fluorescence of the protein was excited in the long-wave absorption spectrum edge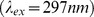, where the contribution of tyrosine residues in the bulk protein fluorescence is negligible. The position and form of the fluorescence spectra were characterized by the parameter 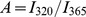, where 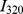 and 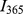 are fluorescence intensities at the emission wavelengths of 320 and 365 nm, respectively [42]. The values of parameter *A* and of the fluorescence spectrum were corrected by the instrument sensitivity. The contribution of the tyrosine residues was characterized by the value 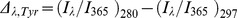. The anisotropy of tryptophan fluorescence was calculated by the equation 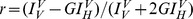, where 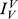 and 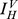
are the vertical and horizontal components of the fluorescence intensity when excited by vertically polarized light and *G* is the relationship of the vertical and horizontal components of the fluorescence intensity when excited by horizontally polarized light 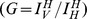, 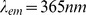 [44]. Specific “green” fluorescence of sfGFP was excited at 365 and 470 nm, and emission was detected at 510 nm.

The unfolding of the protein was initiated by the manual mixing of native or refolded protein solution (50 µl) with a buffer solution (450 µl) containing a needed concentration of guanidine thiocyanate (GTC) or guanidine hydrochloride (GdnHCl). GTC (Fluka, Switzerland) and GdnHCl (Nacalai Tesque, Japan) were used without further purification. The concentration of stock GTC solution and GdnHCl were determined by the refraction coefficient with an Abbe refractometer (LOMO, Russia). The dead time was determined from the control experiments to be approximately 4 s [45], [46]. The GTC dependences of different fluorescent characteristics of sfGFP were recorded following protein incubation in the solution of an appropriate denaturant concentration at 23°C for 1, 24, 45, 69, and 95 h. Refolding of sfGFP was initiated by the dilution of pre-denatured (in 2.2 M GTC) protein (50 µl), with the buffer or denaturant solutions having various concentrations (450 µl). The spectrofluorometer was equipped with a thermostat that held the temperature constant at 23°C.

The recorded fluorescence intensity was corrected to the total optical density of the solution (*D_Σ_*). The corrected fluorescence intensity was defined as 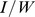, where 
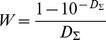
. For details on the correction and normalization of the fluorescence intensity see [47], [48], [49]. These studies show that 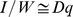, where *D* and *q* are optical density and fluorescence quantum yield of the fluorophore. Only corrected fluorescence intensity can be used for evaluation of the fraction of molecules in the different structural states. After that, the fluorescence intensity was adjusted by the change in optical density with denaturant concentration.

### Circular Dichroism Measurements

CD spectra were obtained using a Jasco-810 spectropolarimeter (Jasco, Japan). Far-UV CD spectra were recorded in a 1-mm path length cell from 260 nm to 190 nm, with a step size of 0.1 nm. Near-UV CD spectra were recorded in a 10-mm path length cell from 320 nm to 250 nm, with a step size of 0.1 nm. Visible CD spectra were scanned from 550 to 320 nm with a step size of 0.1 nm using a 10-mm path length cell. For all of the spectra, an average of 3 scans was obtained. CD spectra of the appropriate buffer solution were recorded and subtracted from the protein spectra. It should be noted that a registration of far-UV CD spectra of protein in the presence of even a small amount of GTC is technically impossible because this denaturing agent is non-transparent for light in this spectral region.

### Stern-Volmer Quenching and Estimation of the Bimolecular Quenching Rates

To evaluate the acceptability of a single tryptophan residue or chromophore of the protein with respect to the solvent molecules, acrylamide-induced fluorescence quenching was studied. Acrylamide (AppliChem, Germany) was used without additional purification. The intrinsic protein fluorescence was excited at 297 and was recorded at 335 nm. The green chromophore fluorescence was excited at 365 or 490 nm and was recorded at 510 nm. The obtained data were corrected based on the solvent signal. The quenching constant was evaluated using the Stern-Volmer equation 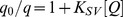, here *K*_SV_ is the Stern-Volmer quenching constant and *Q* is the quencher concentration and subscript 0 indicates the absence of a quencher [50], [51], [52] and, consequently, 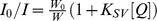. The need to include the ratio of the factor *W*_0_/*W* in the case quencher absorbs at a wavelength of the excitation light, follows from results [47], [48], [49]. The bimolecular quenching rates kq were calculated from 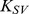 and the mean-square fluorescence lifetime τ according to equation 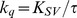 (M^−1^ s^−1^) [50].

### Gel Filtration Experiments

Gel filtration experiments of sfGFP unfolded in GTC were performed on a Superdex-75 PC 3.2/30 column (GE Healthcare, Sweden) using an AKTApurifier system (GE Healthcare, Sweden). Solutions of sfGFP were pre-incubated at 23°C for 24 h in the presence of the desired GTC concentration. Then, 10 µl of this solution was loaded on a column that was equilibrated with the same GTC concentration. The change in the hydrodynamic dimensions of sfGFP was evaluated as a change in the elution volume of sfGFP. In the transition region, the average elution volume of sfGFP was calculated by the equation 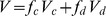, where 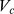 and 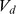 are the elution volumes of the compact and denatured molecules and 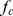 and 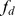
are the portion of compact and denatured molecules estimated as the area of the corresponding peak. A set of proteins with known molecular mass (Chromatography standards from GE Healthcare) were used for plotting a calibration curve.

## Results

The structural features of sfGFP were studied by spectroscopic methods (absorbance, fluorescence, and circular dichroism), tryptophan and green chromophore fluorescence quenching by acrylamide, and size-exclusion chromatography.

**Figure 2:**
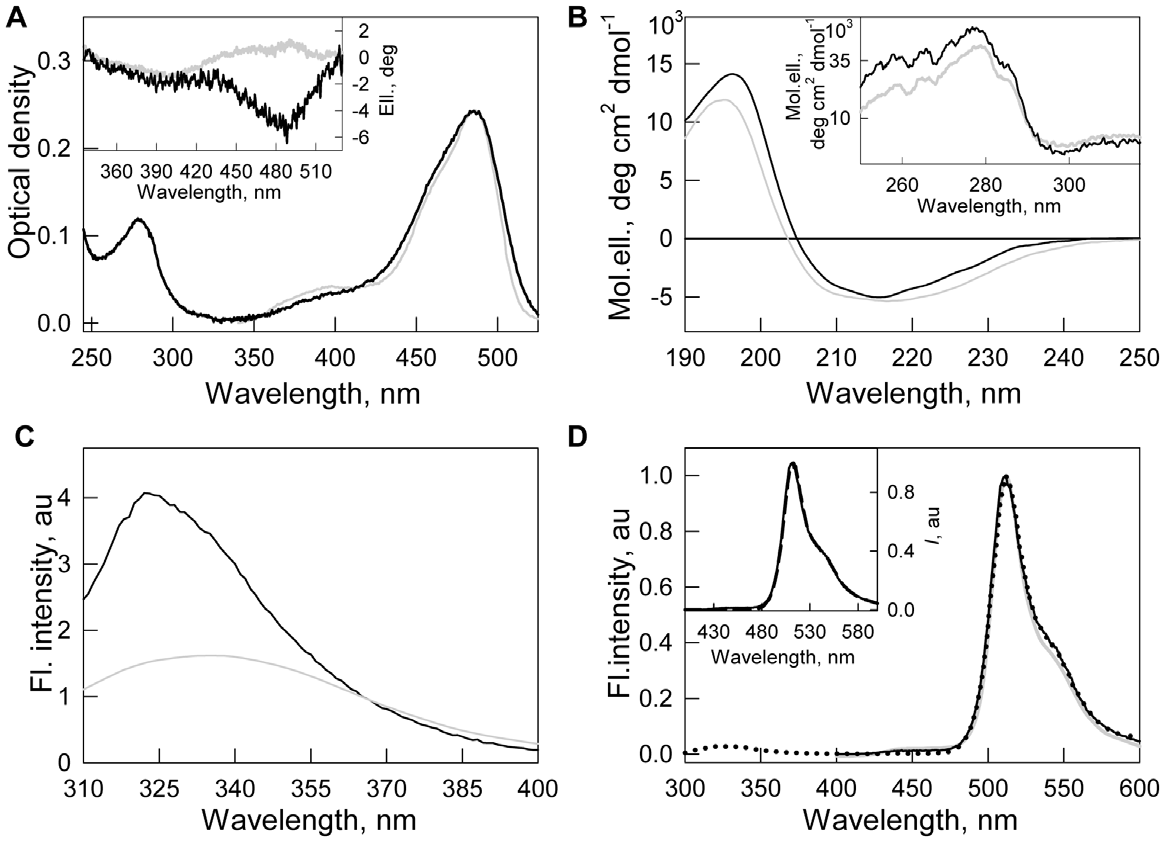
Physicochemical characteristics of sfGFP (black line) in comparison with EGFP (gray line). (***A***) Absorption spectra in UV- and visible spectra regions, (inset to ***A***) CD in visible-UV region. (***B***) CD in far-UV and near-UV (inset to ***B***) regions. (***C***) Intrinsic fluorescence spectra at excitation wavelength of 297 nm. (***D***) Green chromophore fluorescence of both sfGFP and EGFP at excitation wavelength of 390 nm (solid black and gray lines, respectively). Fluorescence spectrum of sfGFP at excitation wavelength of 295 nm (dotted line) is also shown. Fluorescence spectra of sfGFP at excitation wavelengths of 390 nm (solid line), 485 nm (dashed line), corresponding to absorption of neutral and anionic chromophore, are presented on inset to ***D***.

The absorption spectra of sfGFP demonstrated a pronounced band in the UV spectral region, which corresponds to the absorption of the aromatic residues of the protein and the band in the visible spectral region with a maximum at 485 and a shoulder at 390 nm (Fig. 2A). These two peaks in the visible absorption spectra of sfGFP are attributed to the different forms of chromophore, which are discriminated by their ionic states. These are anionic and neutral forms [53], [54]. The far-UV CD spectrum of sfGFP has a negative trough at 215 nm and a positive peak near 200 nm, which is characteristic of proteins enriched with β-sheet secondary structure (Fig. 2B). The near-UV CD spectrum of sfGFP is characterized by a marked positive band (Fig. 2B, inset). The visible CD spectrum of sfGFP has two clearly distinguishable negative bands at 390 and 485 nm (Fig. 2A, inset).

The fluorescence spectrum of a single tryptophan residue of sfGFP in the native state has a peak at 322 nm (Fig. 2C). In addition to the main maximum, a small shoulder at 335 nm is observed in the tryptophan fluorescence spectrum of sfGFP that is in good agreement with the results given in [29]. The fluorescence spectra of the chromophore of sfGFP excited at two wavelengths, 390 and 485 nm, coincide and have a maximum at 512 nm (Fig. 2D, inset). The green chromophore fluorescence can be efficiently excited at 295 nm at the absorption band of the tryptophan residues (Fig. 2D, dotted line), pointing out the existence of an effective non-radiative energy transfer from a single tryptophan residue of sfGFP to the chromophore in neutral form.

The values of the bimolecular constants of tryptophan and chromophore fluorescence quenching by acrylamide (Table 1) indicate that both of them are screened from the solvent, especially green chromophore. The values of the bimolecular constants of green chromophore fluorescence quenching are even lower than that of single tryptophan residue (Trp 48) of azurin [55]. Like FPs, azurin belongs to β-barrel proteins and its Trp 48 is located in the inner cavity of β-barrel scaffold formed exclusively by hydrophobic amino acid residues [43].

**Table 1:** Characteristics of intrinsic and chromophore fluorescence of sfGFP in native and unfolded states.

Parameter	sfGFP in native state	sfGFP in unfolded state (in presence of 2.5 M GTC)
	*Intrinsic fluorescence*	
λ_max_, nm (λ_ex_ 297 nm)	322 (+ a shoulder at 335 nm)	350
Parameter *A* (λ_ex_ 297 nm)	3.9	0.48
*r* (λ_ex_ 297 nm, λ_em_ 365 nm)	0.19	0.09
*τ*, ns (λ_ex_ 297 nm, λ_em_ 335 nm)	0.67	2.46
	*Chromophore fluorescence*	
λ_max_, nm (λ_ex_ 365 nm)	512	–
*I*_max_, %	100	0
*τ*, ns (λ_ex_ 365 nm, λ_em_ 510 nm)	3.32	–
*τ*, ns (λ_ex_ 490 nm, λ_em_ 510 nm)	2.49	–
	*Quenching by acrylamide*	
*k*_q_ of Trp 57 at λ_em_ 335 nm, 10^9^ M^−1^ s^−1^	0.91±0.08	6.1±0.4
*k*_q_ of Cro at λ_ex_ 365 nm, 10^9^ M^−1^ s^−1^	0.06±0.01	–
*k*_q_ of Cro at λ_ex_ 490 nm, 10^9^ M^−1^ s^−1^	0.04±0.01	–

**Figure 3:**
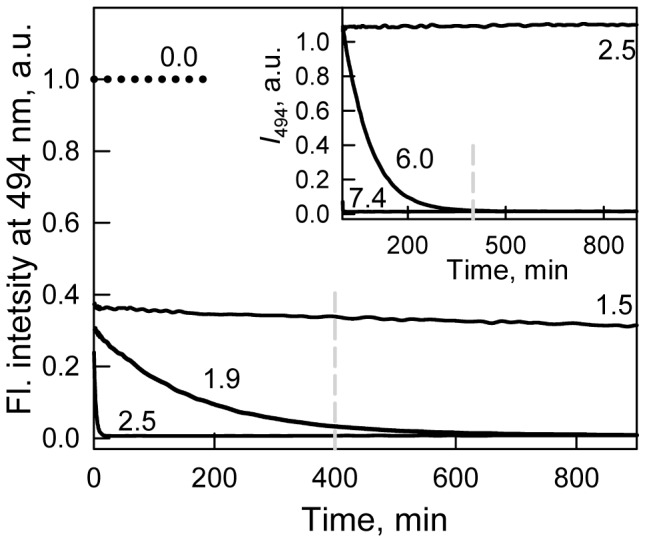
The rate of sfGFP unfolding induced by different denaturing agents. The influence of GTC (*main panel*) or GdnHCl (*inset*) was recorded by chromophore fluorescence intensity at 494 nm. 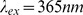. Numbers on the curves are final denaturant concentrations in protein samples. The dashed vertical indicates the time at which the values of chromophore fluorescence intensity were compared.

FPs are known to be highly resistant against denaturing actions [32], [34], [56]. Denaturation of EGFP or sfGFP induced by guanidine hydrochloride (GdnHCl) achieves quasi-equilibrium after several days of protein incubation in the presence of this denaturing agent [29]. Figure 3 shows the kinetics of the denaturation of sfGFP induced by GdnHCl and guanidine thiocyanate (GTC), as recorded by green chromophore fluorescence. In the presence of 1.9 M GTC, green chromophore fluorescence of sfGFP drops to zero in 400 minutes after the addition of denaturant, while to reach the same state, a 3 times higher GdnHCl concentration is required. Because GTC is a stronger denaturant than GdnHCl, we used it in the following experiments.

**Figure 4:**
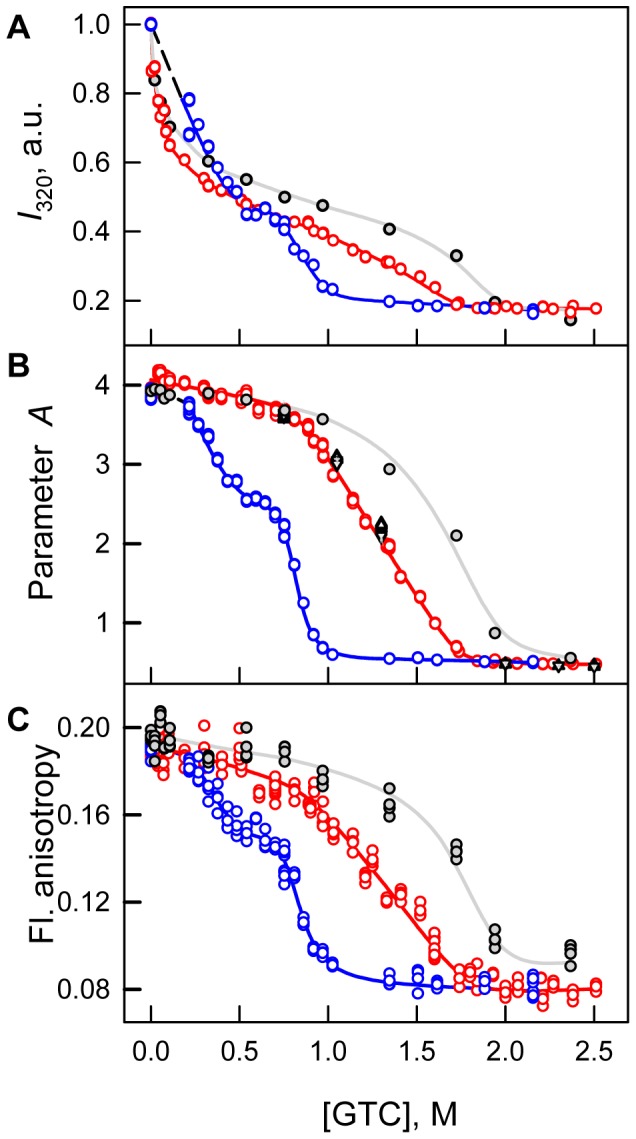
Conformational changes of sfGFP induced by GTC. (***A***) Changes in fluorescence intensity recorded at 320 nm. (***B***) Changes of parameter *A*. (***C***) Changes of fluorescence anisotropy at emission wavelength of 365 nm. 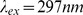. Red circles and line indicate unfolding and blue circles and line represent refolding. Measurements were performed after 24 h incubation of native or denatured protein in the GTC presence. The values of parameter *A* of sfGFP measured after incubation of native protein in the GTC presence during 1 h (gray circles and line), 45 h (black triangles), 69 h (reversed gray triangles) and 94 h (gray crosses) are also indicated.

**Figure 5:**
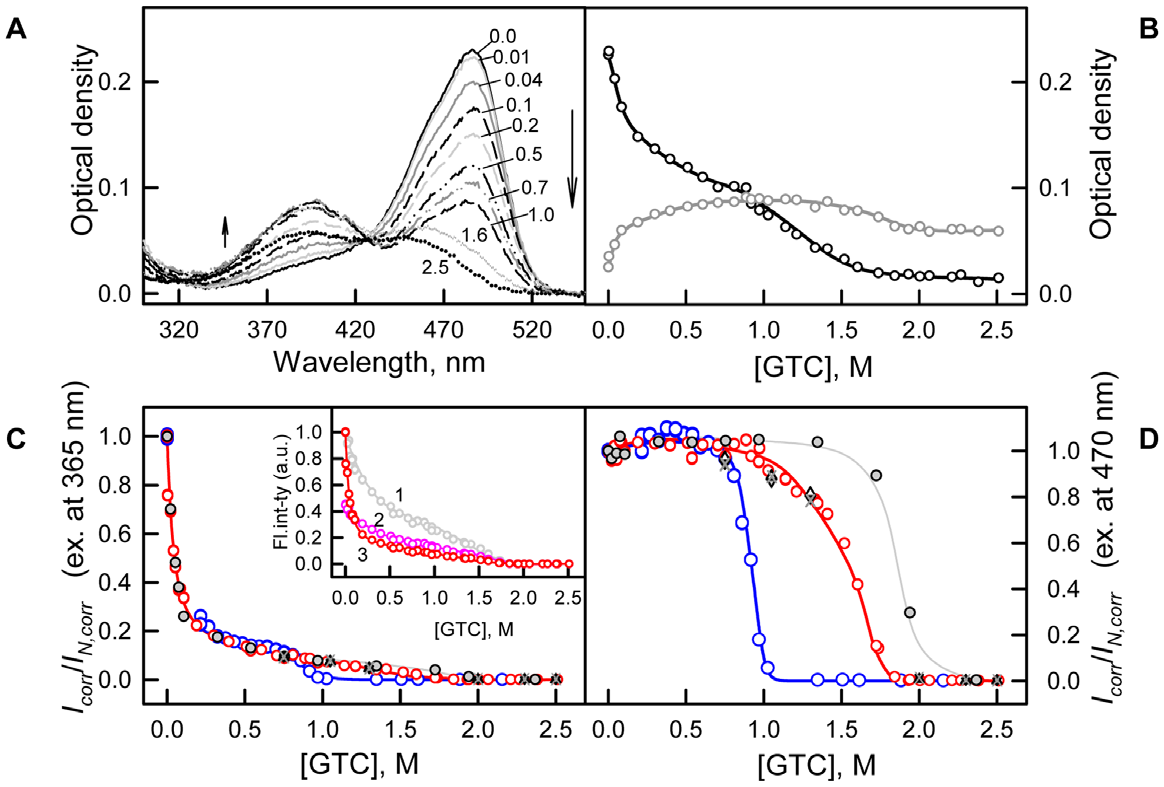
Conformational changes of sfGFP induced by GTC. (***A***) Changes of visible absorption spectra at increasing of GTC concentration as shown by arrows. Numbers at the curves indicate the denaturant concentration. (***B***) Changes of absorbance at 390 nm (gray circles and line) and 490 nm (black circles and line). (***C*** and ***D***) Changes of corrected fluorescence intensity of green chromophore at two wavelengths of excitation of 365 nm and 470 nm corrected to the change of chromophore absorption spectra. Red circles and line indicate unfolding, whereas blue circles and line represent refolding. Measurements were performed after 24 h incubation of native or denatured protein in the GTC presence. The values of chromophore intensity of sfGFP measured after incubation of native protein in the GTC presence during 1 h (gray circles and line), 45 h (black triangles), 69 h (reversed gray triangles) and 94 h (gray crosses) are also indicated. Inset to panel ***C***: experimentally recorded (curve 1, gray), corrected to total density of solution as I/W, where 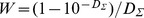 (curve 2, pink), and corrected to the change of chromophore absorption spectra (see, panel **C**) on GTC concentration (curve 3, red) [47], [48], [49].

The denaturation curves for different parameters of sfGFP tryptophan fluorescence (fluorescence intensity, parameter *A* and fluorescence anisotropy; excitation at 297 nm) and sfGFP green chromophore fluorescence intensity (excitation at two wavelengths, 365 and 470 nm; registration at 510 nm) as a function of the final GTC concentration were recorded after 1 to 94 h of equilibration of the protein in the presence of a desired denaturant concentration (Fig. 4 and 5). Quasi-equilibrium curves of GTC-induced unfolding of sfGFP were obtained after 24 hours. Further incubation of sfGFP in the presence of GTC, at least up to 4 days, does not result in measurable changes in the recorded characteristics.

**Figure 6:**
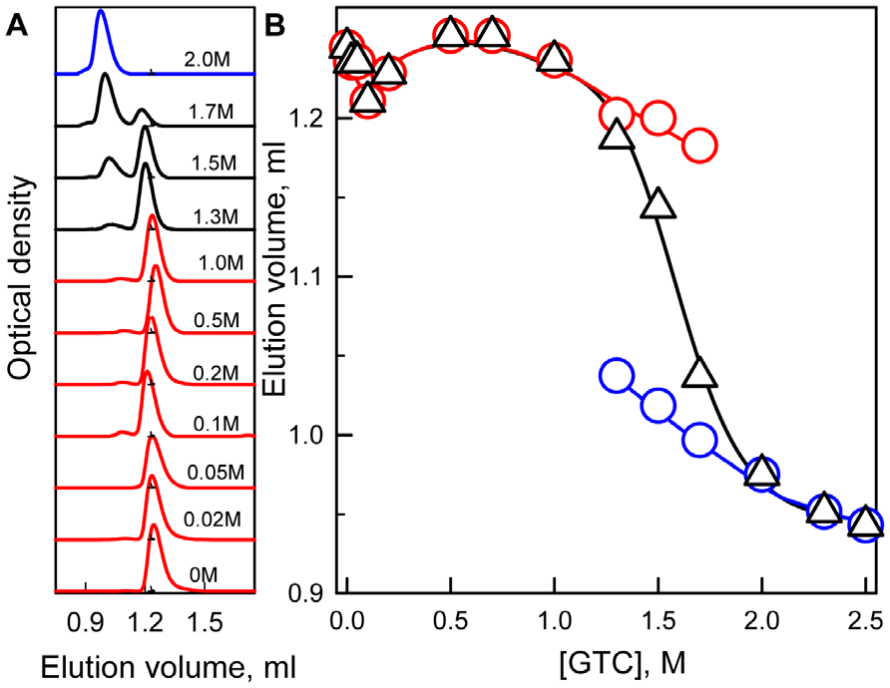
Changes of hydrodynamic dimensions of sfGFP induced by GTC. (***A***) Changes of elution profile of sfGFP at increasing denaturant concentration. Numerals at the curves specify applied denaturant concentration. (***B***) Changes of the position of elution peaks of compact and denatured molecules (red and blue circles, respectively) and the change of averaged elution volume of sfGFP (black triangles). The value of averaged elution volume (<*V*>) was calculated as 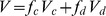, were *f_c_*_(*d*)_ is portion of compact (denatured) molecules and *V_c_*_(*d*)_ is elution volume of molecules in these states. The value of *f_c_*_(*d*)_ is estimated as 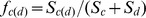, were *S_c_*_(*d*)_ represents the area under peak corresponding to compact (denatured) molecules.

The quasi-equilibrium curve of sfGFP denaturation recorded by tryptophan fluorescence intensity at 320 nm demonstrates that there are two sub-ranges of GTC concentrations in which the behavior of the parameter differs (Fig. 4A). At the small denaturant concentrations (up to 0.7 M GTC), an increase in the GTC concentration is accompanied by a sharp decrease in the value of *I*_320_. At a higher GTC concentration, the trend of *I*_320_ is a sigmoid. The denaturation curves of sfGFP recorded by the change of parameter *A* and fluorescence anisotropy are obviously sigmoid (Fig. 4B and C). As for many other proteins [56], a small increase in the value of parameter *A* is observed in the range of 0.0–0.1 M GTC, suggesting a subtle change in protein structure that is also seen in the shift of elution profile at the same concentrations of GTC (Fig. 6). We explained this affect by elimination of some tensions that are in the protein structure [56]. In the range of GTC concentrations 0.1–0.7 M a monotonous decrease of recorded parameters is observed that is typical behavior for protein characteristics before protein unfolding [57]. A further increase in the GTC concentration results in a drop in the parameter *A* and the fluorescence anisotropy to a value that is typical for proteins in an unfolded state (Fig. 4B and C, Table 1).

Visible absorption spectrum of sfGFP changes dramatically over all the range of GTC concentrations (Fig. 5A and B). But the most pronounced alterations are recorded in the range from 0 to 0.7 M GTC (Fig. 5A and B). Here, the visible absorption spectra of sfGFP demonstrated a pronounced drop in the absorption band at 485 nm, which corresponds to the anionic form of the chromophore, with a concomitant rise in the absorption band at 390 nm, which corresponds to the neutral chromophore. Further changes in the intensity of both of the absorption bands can be described as sigmoid curves. Some blue shift of the absorption band at 485 nm is observed at a GTC concentration exceeding 1.3 M. An optical density at approximately 425 nm remains unaltered in all range of GTC concentrations (Fig. 5A). The presence of such an isosbestic point in the visible absorption spectra of sfGFP indicates that only two types of molecules namely sfGFP molecules with neutral and anionic chromophore forms exist and the observed alterations of the visible absorption spectra are caused by changes in the ratio between them.

Changes depending on the GTC concentrations followed by sfGFP green chromophore fluorescence intensity at wavelengths of excitation 365 (see inset to Fig. 5C, curve 1) and 470 nm (data are not shown) are of the same character as observed for the *I*_320_ curve. To account for the changes in optical density, we corrected the denaturation curves of the green chromophore fluorescence intensity at excitation wavelengths of 365 and 470 nm according to the changes in the absorbance of sfGFP at these wavelengths as it is described in the 'Materials an methods' section (Fig. 5C and D). The response to small GTC concentrations of corrected fluorescence of green chromophore excited at the two wavelengths is completely different. Excitation at 365 nm results in a significant drop of fluorescence quantum yield (up to 13% of the native protein signal, Fig. 5C), whereas no apparent changes in the corrected fluorescence of green chromophore with respect to native sfGFP are seen at excitation at 470 nm (Fig. 5D).

Structural changes of sfGFP during denaturation were also characterized using gel filtration (Fig. 6). These experiments were performed with a Superdex75 PC 3.2/30 column. Native sfGFP elutes as a single peak, which has an elution volume that corresponds to a protein with a molecular mass of 32.4 kDa (Fig. 6A). This value is in excellent agreement with the molecular mass of sfGFP, as estimated from its amino acid sequence and accounting for the weight of His-tag (30.1 kDa). At moderate denaturant concentrations (below 1.0 M GTC), sfGFP still elutes as a single peak with an elution volume that corresponds to compact molecules. The position of this elution peak changes only slightly with respect to that of the native protein. When the GTC concentration is increased to higher than 1.0 M, a second elution peak appears, with an elution volume that corresponds to molecules that have a less compact structure. The intensity of this peak rises with the GTC concentration, and at 2.0 M GTC, only a second peak remains in the sfGFP elution profile. The analysis of elution profiles of sfGFP at different GTC concentrations allowed for calculating an average elution volume of sfGFP, which characterizes the protein’s hydrodynamic dimensions averaged over all conformations of the protein. The GTC dependence of the average elution volume of sfGFP is sigmoid (Fig. 6B), but a local minimum at 0.1 M GTC can be observed. A transition from the more compact to the less compact molecules occurs in the range of GTC concentrations of 1.0–2.0 M.

Renaturation of sfGFP was induced by the dilution of the pre-denatured protein in 2.2 M GTC to the various final denaturant concentrations (Fig. 4, 5). Refolding of sfGFP reaches equilibrium after 24 hours of protein incubation at appropriate GTC concentrations. Under strongly refolding conditions (the final concentration of GTC is 0.22 M), all of the recorded characteristics of sfGFP recover to the level of the native protein, which indicates the reversibility of protein unfolding (Fig. 4, 5). Furthermore, the dependences of unfolding of refolded protein recorded by the change of parameter *A* and fluorescence anisotropy coincide with that recorded for native protein, that also proves the reversibility of protein unfolding. However, the value of *I*_320_ at low GTC concentrations measured for sfGFP on the refolding pathway even exceeds the value of the parameter determined for sfGFP on the unfolding pathway at the same denaturant concentrations (Fig. 4A). The unfolding and refolding curves of sfGFP in the transition area do not match, hence showing apparent hysteresis. The refolding curves of parameter *A* and fluorescence anisotropy of sfGFP exhibit the plateau in the range of 0.5–0.7 M GTC, and the values of these parameters are well below the values measured at protein denaturation (Fig. 4B and C). It is interesting that the fluorescence intensity of the sfGFP green chromophore recorded during the denaturation and renaturation experiments coincides in this range of GTC concentrations (Fig. 5C and D).

## Discussion

**Figure 7:**
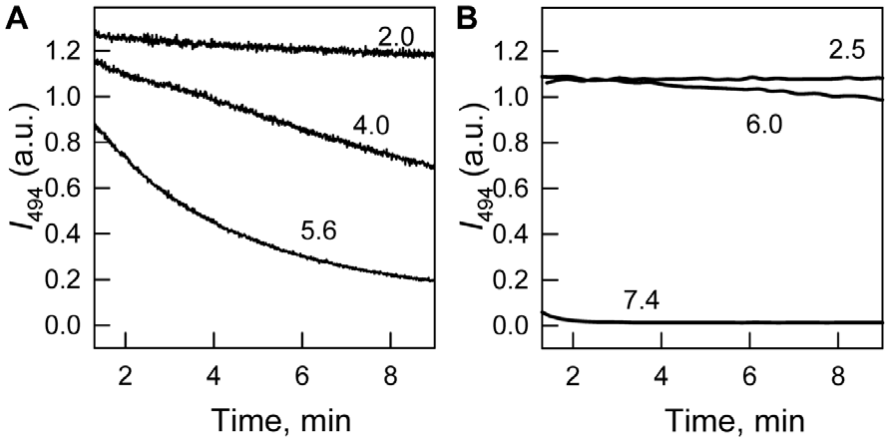
Kinetics of GdnHCl-induced unfolding of EGFP (*A*) and sfGFP (*B*). The changes of chromophore fluorescence intensity at 494 nm during first 10 min of protein unfolding are shown. 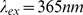. Numbers at the curves indicate the denaturant concentration.

sfGFP, having six additional substitutions of amino acids over the 'cycle-3' GFP variant and nine substitutions of amino acids in comparison with EGFP (Fig. 1), was developed to exhibit strong folding efficiency. It also possesses faster folding kinetics [34], [58] and higher denaturant resistance compared to EGFP, as revealed by comparative studies of GdnHCl-induced denaturation (Fig. 7). Surprisingly, despite of numerous EGFP applications, its spatial structure was determined only recently [39]. Although all but one (Phe145) of these amino acid substitutions are solvent-exposed and located far from a tryptophan residue or chromophore of sfGFP (Fig. 1), we wondered whether they could possibly affect the spectral or structural properties of sfGFP.

### Spectral Properties of sfGFP in Native State

The absorption spectra of sfGFP and EGFP in the visible spectral region are quite similar (Fig. 2A). The two-humped shape of the spectra of both proteins in this spectral region is determined by the presence (in solution) of the protein molecules bearing chromophores in neutral or anionic forms [20], [53]. For sfGFP in the native state, the occurrence of these two forms of chromophores was also demonstrated by nuclear magnetic resonance [30]. The intensity of the two maxima, at 390 and 490 nm, in the protein absorption spectra is defined by the portion of molecules with neutral or anionic chromophores. EGFP has a more pronounced absorption peak corresponding to neutral chromophore in comparison to that of sfGFP. We can suggest that substitution of Tyr 145 located nearby to green chromophore in EGFP with Phe 145 not having hydroxyl group could influence on internal proton network and thus change the distribution between the protein molecules having chromophore in the neutral or anionic forms in sfGFP.

According to its elution profile, sfGFP in its native state is present in solution as a monomer (Fig. 6A). The visible CD spectrum of sfGFP in its native state is characterized by two negative bands, at approximately 390 and 490 nm, which look like a mirror reflection of its absorption spectrum (Fig. 2A, inset). We suppose that the complex shape of the visible CD spectrum of sfGFP in its native state arises from the contribution of neutral and anionic chromophores of sfGFP. It cannot be excluded that the difference in the intensities of the two bands of the protein visible CD spectrum can be attributed to the diversity of the portion of the protein molecule with the neutral or anionic chromophore. Nevertheless, the effect of circular dichroism arises from the chirality of both the chromophore and its nearest environment and can thus be influenced by local structural differences around the chromophore when it is in the anionic or neutral forms.

**Figure 8:**
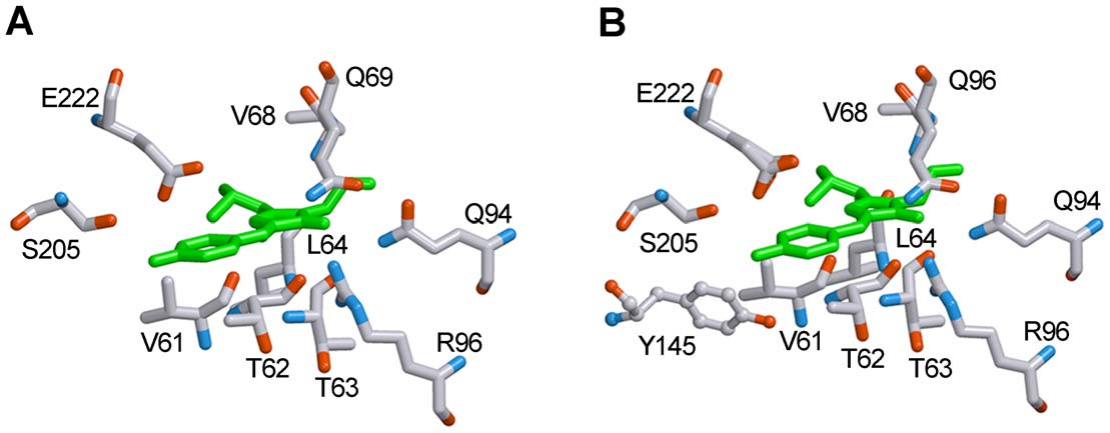
The structure of chromophore environment of sfGFP (*A*) and EGFP (*B*). The localizations of residues involved in chromophore environment are shown. Tyr145 near the chromophore of EGFP (***B***), which is absent in the environment of sfGFP (***A***), is drawn in a distinct manner. Carbon, nitrogen and oxygen are gray, blue and red, respectively. The figure is created on the basis of PDB data [38] with the files 2B3P.ent for sfGFP [35] and 2Y0G.ent for EGFP [39], using the graphical software VMD [69] and Raster 3D [70].

It is interesting that the visible CD spectrum of EGFP also contains two extremes, but they have opposite signs (Fig. 2A, inset). A negative band at 390 nm of the visible CD spectrum of EGFP almost coincides with that of sfGFP, whereas a positive band at 490 nm does not match the corresponding spectral region of sfGFP. This could indicate diversity in the structure around the anionic chromophore that is involved in the two proteins because only the anionic chromophore is responsible for the spectral properties of both of the proteins in this spectral region. Analyzing the crystallographic data of both proteins, the only difference that we observed is the presence of Tyr145 near the chromophore of EGFP [39], which is substituted with Phe145 in the chromophore environment of sfGFP [35] (Fig. 8). Conceivably, the proximity of this residue to the chromophore in EGFP could not only disturb the chromophore ionization but greatly affects the spectral properties of the anionic chromophore.

The far-UV CD spectrum of sfGFP is similar to that of EGFP although it shows a more pronounced positive peak (Fig 2B). The deconvolution of the far-UV CD spectrum of sfGFP using Provencher’s algorithm [59] revealed approximately 11% α-helices, 42% β-sheets and 21% β-turns, which is consistent with the crystallographic data [35]. The deconvolution of the far-UV CD spectrum of EGFP yields the same amount of β-turn (21%) as sfGFP but a slightly lower amount of β-sheets (36%) and a higher amount of α-helices (16%). The higher amount of ordered β-structure of sfGFP in solution compared to EGFP could arise from the stabilization of the β-barrel through expanding the contacts between the amino acid side chains. In the sfGFP, mutant residue Arg30 forms an extensive network of electrostatic interactions and additional hydrogen bonds that involve residues Glu32– Arg30– Glu17– Arg122– Glu115 from the four adjacent β-strands, whereas the side chain of the original residue Ser30 is only hydrogen bonded to Glu17 [35].

The thryptophan fluorescence spectrum of sfGFP in its native state is characterized by a complex shape with main short-wave maxima and additional shoulder that is red shifted in the wavelength scale (Fig. 2C). Because sfGFP has only one tryptophan residue in position 57, its fluorescence properties are defined by this residue and its microenvironment. Usually, blue-shifted spectra are typical of tryptophan residues that are in microenvironments mainly formed by hydrophobic residues, as, for example, it is in the case of single tryptophan residue Trp 48 of azurine [43] which has a unique blue fluorescence spectrum with maximum at 308 nm. Another explanation of such fluorescence properties is a highly rigid microenvironment of a tryptophan residue even if it is composed by predominantly polar residues [40], [60]. An analysis of peculiarities of the location and characteristics of the microenvironment of Trp57 in sfGFP using a previously described scheme [40] showed that, in sfGFP, the second scenario is realized. The density of the microenvironment of Trp57 of sfGFP is high (there are as many as 81 atoms in a 7 Å radius from the indole ring of Trp57, as shown in Table 2). In comparison, there are only 69 atoms the microenvironments of Trp 48 of azurin [43] and 78 and 69 atoms the microenvironments Trp340 and Trp356 of the actin, which are responsible for its blue-shifted fluorescence spectrum with a maximum at 325 nm [60]. Thus, the microenvironment of Trp57 in sfGFP is likely to be rigid. This conclusion is also supported by the pronounced near-UV CD spectrum of sfGFP in its native state (Table 3, Fig. 2B, inset). The experimental data on the quenching of tryptophan fluorescence by acrylamide show a low accessibility of Trp57 to solvent molecules (Table 1), which further proves the high density of its microenvironment. The Trp57 microenvironment contains several atoms of polar amino acids (Table 3). Except for these atoms, there are 2 aromatic residues, such as Phe46 and Tyr143, and 2 proline rings of Pro56 and Pro58 near the tryptophan residue. Large aromatic and proline rings could restrict the motion of Trp57, which is in agreement with the high value of the fluorescence anisotropy of sfGFP in its native state (Table 1).

**Table 2:** Side chain conformation of Trp57 in sfGFP.

N (*d*)*	*χ*_1_, (deg)*	*χ*_2_, (deg)*
81 (0.77)	277	107

* N is the number of atoms in the microenvironment of tryptophan residue; *d* is the density of tryptophan residue microenvironment; *χ*_1_ and *χ*_2_ are the angles characterizing the conformation of tryptophan residue side chain.

**Table 3:** Characteristics of the Trp57 microenvironment in sfGFP.

*N*	*residue*	*atoms*	*R* *, *Å*
1	Asp216	OD1(NE1)	2.82
2	Asp216	OD2(NE1)	4.12
3	Lys209	NZ(CD1)	3.91
4	Met218	SD(CE3)	4.18
5	Tyr143	OH(CG)	5.31
6	Cys48	SG(CZ2)	5.65
7	HOH	(CD1)	3.57
8	HOH	(CD1)	3.67
9	HOH	(CD1)	3.95
10	HOH	(NE1)	4.21
11	HOH	(CD1)	3.90
12	HOH	(NE1)	5.32
13	Phe46	CG	4.62
14	Tyr143	CE1	4.45
15	Pro56	CA	5.45
16	Pro58	CD	4.8

* *R* is the minimal distance between a residue involved in the microenvironment of tryptophan residue and an indole ring.

Residues 1–12 are polar groups of the Trp57 microenvironment.

Residues 13–16 are aromatic residues of the Trp57 microenvironment.

A heterogeneity of Trp57 for sfGFP [30] and ECFP [61] has been recently shown using nuclear magnetic resonance. This heterogeneity can be attributed to the *cis*- and *trans*-conformations of the peptide bond between the Trp57 and Pro58 residues [61]. Such isomerization around this peptide bond can result in changes in distances between the atoms of the indole ring of Trp57 and the atoms of its microenvironment, which thereby alter the properties of the tryptophan microenvironment. We assume that the complex spectra of tryptophan fluorescence of sfGFP can arise from the presence of protein molecules that differ in conformation at the Trp57– Pro58 peptide bond.

In contrast to sfGFP the tryptophan fluorescence spectrum of EGFP is obviously red-shifted and quenched (Fig. 2C). The most probable reason of these dramatic difference is the change of Tyr 145 in EGFP [39] to Phe 145 in sfGFP [35]. The near-UV CD spectrum of EGFP is similar to that of sfGFP, although it is less intense (Fig. 2B, inset). This can be connected with the difference in the absorption spectrum of these two proteins.

### Different Action of GTC on sfGFP Structure

To study the processes of sfGFP unfolding – refolding we used GTC, which is a stronger denaturing agent compared to GdnHCl (Fig. 3). This choice allowed us to reach equilibrium unfolding of all of the registered characteristics of sfGFP in a short time (Fig. 4 and 5), thereby avoiding possible artificial data that could be attributed, for example, to oxidative processes of the green chromophore during the prolonged incubation of the unfolded protein in denaturant.

To follow the structural changes of sfGFP, we increased the denaturant concentration from 0 to 2.5 M. In the presence of 2.5 M GTC, sfGFP is in an unfolded state, as indicated by the values of all of the registered characteristics of the protein (Table 1). At these conditions green fluorescence of sfGFP is completely quenched which is indicative of protein structure disruption [26]. A red shift in the tryptophan fluorescence spectra and a decrease in its quantum yield suggest an increase in accessibility of the tryptophan residue to molecules of solvent (Table 1). This is also confirmed by the increase in the bimolecular quenching constant to a value that is typical of free tryptophan in water (Table 1). The sfGFP denaturation is followed by an increase in tyrosine fluorescence that results from the increase in the distances between the tyrosine and tryptophan residues, which prevents an effective energy transfer between them (data are not shown). Notably, the value of an excited state lifetime of tryptophan fluorescence of sfGFP in an unfolded state is larger than that of sfGFP in a native state (Table 1). We assumed that Trp57 is quenched by an energy transfer to green chromophore when it is incorporated in the native globule of sfGFP (Fig. 2D). In the denatured protein, there are no conditions for the effective energy transfer from the tryptophan residue to a chromophore, which results in an increase in the fluorescence lifetime (Table 1).

The behavior of all of the recorded parameters, including the tryptophan and green chromophore fluorescence of sfGFP, the chromophore absorption bands in its neutral and anionic forms as well as the average elution volume of sfGFP depending on the GTC concentration, allows us to distinguish three overlapping concentrations of GTC, in which denaturant has a different effect on the protein (Figs. 4, 5, 6). In the concentration range from 0 to about 0.1 M GTC small structural changes of the protein are shown in a slight increase in the parameter *A* (Figure 4 B) and the anisotropy of tryptophan fluorescence (Figure 4 C), and change of the position of the elution peak in experiments on gel permeation chromatography (Figure 6). In the entire investigated concentration changes of GTC change of the absorption spectrum of the protein is revealed (Figure 5 A and B). At the same time, in the concentration 0 to 0.7 M GTC there is a significant decrease in the intensity of tryptophan fluorescence (Figure 4 A) and chromophore fluorescence excited at 470 nm (data are not shown). This is caused by decrease in the fraction of sfGFP molecules in the anionic form of the chromophore and, thus, decreasing the optical density of the solution at the wavelength of the excitation light rather than structural transitions (Figure 5 A and B). The confirmation of this is the character of the dependence of the parameter *A* (Figure 4 B), the anisotropy of tryptophan fluorescence (Figure 4 C), and the corrected fluorescence intensity (Figure 5 D). The dependence of the fluorescence intensity of the chromophore (510 nm) excited at the short-wavelength band of sfGFP absorption (365 nm) differs significantly from that excited at the long-wavelength band (Figure 5 C and D). It is known that the excitation of a neutral chromophore is followed by its deprotonation, with the formation of an exited state of anionic chromophore I^*^, and subsequent emission from this state [62]. We suppose that complex behavior of fluorescence intensity of sfGFP recorded at 510 nm and exited at 365 nm is caused by two reasons, by an increase of the fraction of sfGFP molecules with neutral form of the chromophore, as well as by the decrease in the equilibrium constant of transition from neutral to anionic form in the in the excited state with increasing concentration of GTC. The increase in concentration of sfGFP molecules with neutral chromophore also explains the sharp decrease in the intensity of tryptophan fluorescence due to energy transfer from Trp to the chromophore in the neutral form. In the denaturant concentration from 0.7 to 1.7 M GTC, sfGFP molecules unfold (Figures 4, 5, 6), as, apparently, "all or nothing" process.

All of the observed effects at small GTC concentrations are probably caused by negatively charged ions of thiocyanate. Such an effect was previously observed for several yellow FPs, such as eYFP [54], [63] and E2GFP [64], in the presence of different anions (chloride, halogens, nitrates and thiosulfates). In the case of eYFP, a specific binding site has been shown for anions located near the chromophore and Gln69. In contrast, the halogen binding site of E2GFP is positioned over the chromophore and near the residue Tyr203. Inhibition of anionic chromophore formation is expected to occur in the presence of a negatively charged anion near the chromophore. To our knowledge we are first who observed low denaturant effects on green FP characteristics which are similar to changes of characteristics of yellow FP variants induced by different anions.

Another possibility of the mentioned effect at small GTC concentrations can be attributed to an influence of the positively charged ions of guanidine, GdnH^+^. For several proteins, such as creatine kinase [65], actin [45], [66], carboanhydrase [66], [67] and odorant-binding protein [51], it has been shown that interactions of GdnH^+^ ions with the carboxyl groups of glutamic and aspartic amino acids and the amide groups of glutamine and asparagine at the protein surface neutralized the negatively charged protein regions. These resulted in the reduced local structural tensions of the protein globule and a stabilization of the whole protein molecule or an aggregative effect on the protein structure. However, this question should be addressed more deeply to find out what type of processes underlie the sfGFP structural changes that occur at low GTC concentrations. Notably, in studies of FPs where ionic denaturants are applied their possible influence on FP characteristics should be considered.

The disruption of the protein structure at GTC concentrations above 0.7 M is manifested by simultaneous changes in all of characteristics of sfGFP, such as the tryptophan fluorescence intensity, parameter *A*, the fluorescence anisotropy, the green chromophore fluorescence intensity and the hydrodynamic dimensions of the protein (Fig. 4, 5, 6). Changes in the absorption spectra in the visible spectral range of sfGFP at high GTC concentrations also correlate with protein unfolding (Fig. 5A and B). An apparent blue shift in the absorption spectra of the anionic chromophore form of the green fluorescent proteins can be attributed to destabilization of the anionic chromophore in its exited state in the course of protein denaturation [20], [53]. It is interesting that FP mutants defective on chromophore formation are characterized by lowered stability with respect their chromophore bearing counterparts [29], [56], [68]. For example, sfGFP mutants defective of chromophore synthesis are a quarter resistant to GdnHCl as compared to sfGFP [29]. We suppose that chromophore interactions with β-barrel matrix surrounding it contribute appreciably to high protein stability.

### Reversibility of GTC-induced Denaturation of sfGFP

GTC-induced denaturation of sfGFP is reversible, as confirmed by the recovery of all of the recorded parameters of the protein to the same level as the native protein at decreasing denaturant concentrations (Fig. 4, 5). An apparent hysteretic behavior of the folding and unfolding curves of sfGFP is presumed to be connected with the presence of the green chromophore, which must be locked into the correct active form in the protein core at the latest folding step [29]. Indeed, unable to form chromophore mutant variants of GFP showed no hysteresis of the unfolding and refolding curves [29]. Obviously, hysteretic behavior of unfolding-refolding pathways is a distinguishing feature of FPs which are able of forming chromophore. Notably, in the range of 0.5–0.7 M GTC, the values of parameter *A* and the fluorescence anisotropy of sfGFP recorded during the protein refolding do not match the values that are obtained during protein unfolding and are significantly lower (Fig. 4B and C). At the same time, the green fluorescence intensity is already recovered in this denaturant region (Fig. 5C and D). This reflects an accumulation of an intermediate state on the refolding pathway of sfGFP. It has been shown by molecular dynamics simulations that an initial fast formation of the sfGFP β-barrel is followed by a slow search through the chromophore isomerization and structural fluctuations toward a locked active native fold [31]. During the slow second folding step, the incorporation of final β-strands into the barrel and structural rearrangement at the lid of the barrel occur [30], [31]. Because those β-strands are near Trp 57, the structural changes around this region could be emerged by sfGFP tryptophan fluorescence (Fig. 4B and C). Probably the observed intermediate state of sfGFP has an organized core around the green chromophore, whereas the structure around Trp57 is not yet formed. It is worth noting that at GTC concentrations below 0.5 M sfGFP, the fluorescence characteristics of the refolding pathway are averaged over the two protein states, with one state having an un-packed structure and the other state having an already packed structure. While on the unfolding pathway, sfGFP preserves its native state in this GTC range, for which the fluorescence characteristics are influenced by the interaction of protein molecules with denaturant molecules, as we proposed above. This is a possible reason for the notable difference in the sfGFP fluorescence characteristics measured during the protein refolding and unfolding at low denaturant concentrations (Fig. 4).

In conclusion, native folding of sfGFP β-barrel proceeds in the absence of chromophore while at refolding of sfGFP from unfolded state the protein contains the mature chromophore which should be locked inside the β-barrel. This is the reason of hysteretic behavior of unfolding – refolding curves of sfGFP. Once being formed, the chromophore plays a crucial role in protein stability strongly holding the native scaffold of the protein.
